# Conducting Polymer-Based Nanofluidic Membranes for Osmotic Energy Conversion

**DOI:** 10.3390/membranes16090301

**Published:** 2026-09-14

**Authors:** Sinuo Zhou, Chengyang Jia, Ying Zhang, Boyu Sun, Xin Xi, Shuhan Yang, Lipeng Liu, Guoyu Zhang, Xiaoyan Nie, Qiang Wang, Siqi Liu, Yanan Xie, Zhenhang Wang

**Affiliations:** College of Biological and Chemical Engineering, Qilu Institute of Technology, Jinan 250200, China

**Keywords:** conductive polymer, nanofluidic membrane, osmotic energy conversion, reverse electrodialysis, ion permselectivity, polypyrrole, polyaniline

## Abstract

Osmotic energy conversion (blue energy), serving as a sustainable marine renewable energy source, converts Gibbs free energy originating from salt concentration differences into electric power by virtue of ion-selective nanofluidic membranes. Conventional commercial ion-exchange polymer membranes suffer from inherent limitations, including low transmembrane flux, insufficient ion permselectivity, severe interfacial concentration polarization, poor salt tolerance, and unsatisfactory long-term structural stability. These drawbacks greatly restrict the energy conversion efficiency and large-scale engineering application of reverse electrodialysis (RED). Conductive polymers (CPs), mainly including polypyrrole (PPy), polyaniline (PANI), polythiophene (PTh), and their derivatives, possess the distinctive merits of tunable surface charge density and polarity, outstanding electronic conductivity, facile nanochannel structural regulation, and reversible redox responsiveness, making them ideal building blocks for advanced nanofluidic membranes for high-efficiency osmotic energy conversion. This review summarizes recent progress in the fabrication of conductive polymer-based nanofluidic membranes, comprehensively compares the osmotic output performance of typical CP material systems, and discusses the core metrics of osmotic energy conversion output performance. By providing an overview of these developments, this review aims to offer insights into the future development of conductive polymer-based nanofluidic membranes for osmotic energy conversion.

## 1. Introduction

Global carbon neutrality strategies have promoted intensive research on clean renewable energy worldwide. Osmotic energy widely exists at river–sea interfaces, inland salt lakes and industrial brine discharge sites, with enormous theoretical reserves. Reverse electrodialysis (RED) acts as the mainstream technology for osmotic energy conversion [1,2,3,4]. Driven by chemical potential differences between concentrated and dilute salt solutions, directional selective ion transport across charged nanochannels generates continuous ionic current, thus realizing the conversion from osmotic pressure to electric energy. Membrane material properties determine the upper limit of RED system efficiency. Emerging nanofluidic membrane technologies utilize electric double layer (EDL) overlap in nanoconfined channels to achieve precise ion screening, which effectively balances ion permselectivity and transmembrane flux [5,6,7]. The Poisson–Nernst–Planck (PNP) equations, combined with Manning counterion condensation theory, have laid solid theoretical foundations for the numerical simulation of nanofluidic ion transport behaviors [8,9,10,11].

**Figure 1:**
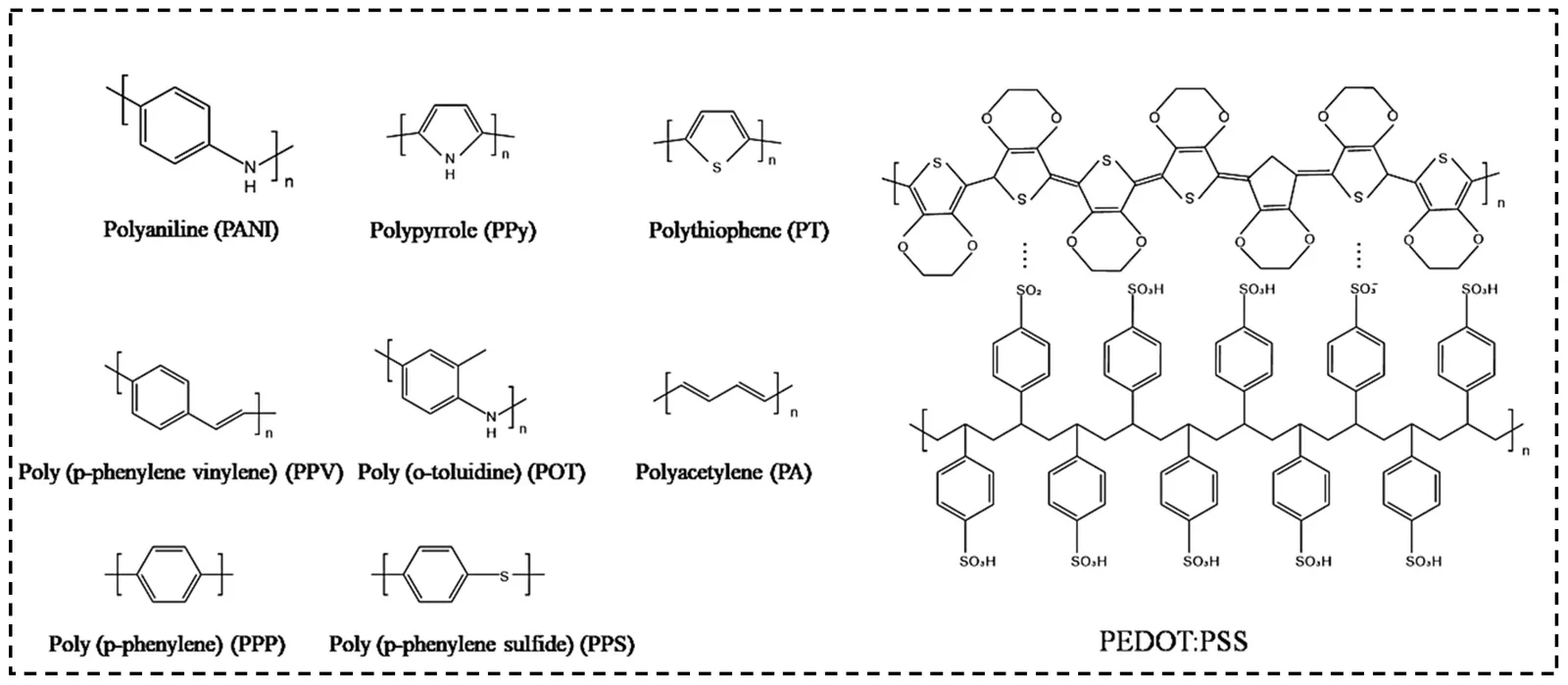
Chemical architectures of some conductive polymers [12].

Conjugated polymers (CPs) feature polymeric backbones with extensive π-conjugated networks, which endow them with metal-like magnetic, electronic, and optical properties. Ever since the discovery of highly conductive polyacetylene (PAc) in the mid-1970s, these materials have remained a central research focus in polymer science [12]. Representative examples include polythiophene (PT), poly(3,4-ethylenedioxythiophene) (PEDOT), polyparaphenylene (PPP), polyaniline (PANI), polypyrrole (PPy), and polyacetylene (PA), whose molecular structures are depicted in Figure 1.

Conductive polymers integrate the excellent processability of organic polymers and semiconductor electronic conductivity [13,14,15]. Different from inert polymeric nanochannels with fixed static surface charges, PPy and PANI can dynamically regulate surface charge density and polarity through doping/dedoping redox reactions, endowing nanochannels with stimulus responsiveness toward pH, applied voltage, and ambient ions [16,17,18]. In situ electrochemical polymerization can construct intact continuous conductive networks inside nanochannels, achieving synergistic ionic–electronic dual conduction and effectively alleviating the universal selectivity–permeability trade-off dilemma faced by most porous charged membranes.

## 2. Principle and Key Factors of Osmotic Energy Conversion

### 2.1. The Basic Principle and the Device

Once a salinity gradient is established, an osmotic pressure difference emerges between solutions of disparate concentrations, driving spontaneous diffusion–osmotic transport powered by Gibbs free energy [19,20]. Solute ions spontaneously migrate from the high-salinity compartment to the dilute side [19]. On this basis, the theoretical osmotic energy can be quantified via the following expression:

(1) $$dG=-\frac{\mathrm{RT}}{F}\ln\frac{\tau_{H}}{\tau_{L}}(|I_{+}|+|I_{+}|)\mathrm{dt}$$

In Equation (1), *R*, *T*, and *F* correspond to the universal gas constant, absolute temperature, and Faraday constant, in sequence. τ denotes the solution activity of electrolytes on the high-salinity (H) and dilute (L) sides, and I corresponds to the diffusion current contributed by cations and anions [21]. It is worth emphasizing that cation and anion diffusion collectively contribute to the total theoretical osmotic energy, yet merely the net current stemming from asymmetric ion diffusion can be extracted as electricity. The degree of asymmetric ion diffusion within bulk solutions relies largely on the distinct migration mobilities of cations and anions [22]. Certain ion combinations, like cation/anion pairs such as K^+^ and Cl^−^ ions, exhibit close bulk mobilities, which leads to an extremely weak net output current.

**Figure 2:**
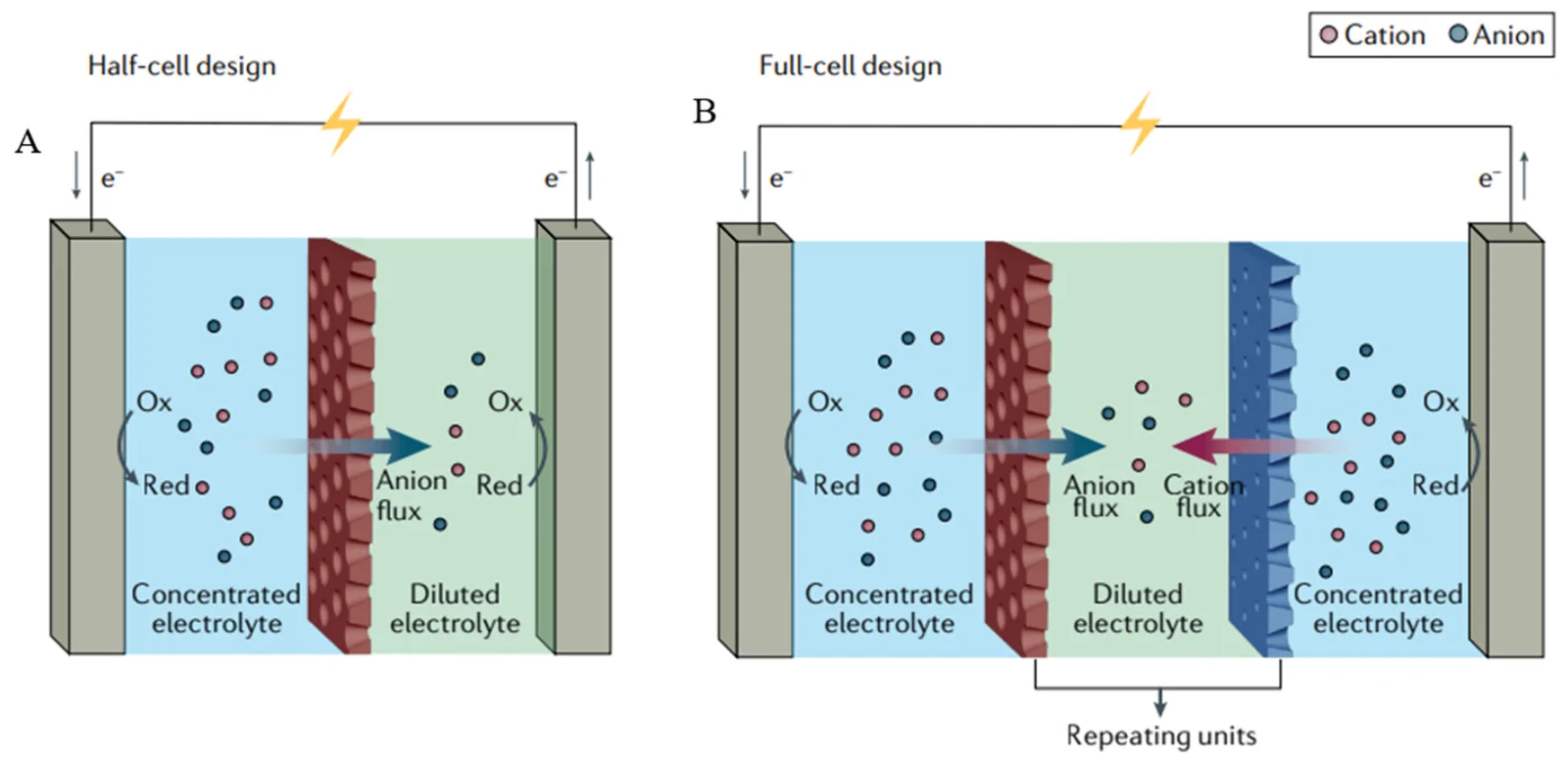
Schematic diagrams of half-cell and full-cell architectures for nanofluidic osmotic energy conversion systems based on a (**A**) half-cell design and (**B**) full-cell system [26].

To address this bottleneck, two categories of reverse electrodialysis-based architectures have been developed for osmotic power harvesting [23,24,25,26]. Figure 2A illustrates a typical half-cell configuration for osmotic energy conversion. This setup consists of two compartments loaded with electrolytes of disparate concentrations, an ion-selective membrane that partitions the two solutions, and a pair of electrode. Owing to the ion-sieving effect of the selective membrane, one ionic species diffuses far faster than its counterpart, greatly boosting the recoverable proportion of osmotic energy relative to pure bulk solution systems. Redox reactions proceed at electrode surfaces, which transfer the net current originating from selective ion permeation to the external circuit while preserving overall electroneutrality inside the device. Benefiting from its high detection sensitivity, compact device structure, and facile operation procedures, the half-cell configuration is widely adopted to investigate fundamental transport mechanisms and screen high-performance ion-selective membranes for osmotic energy recovery [25,26]. Stacking alternating cation-selective and anion-selective membrane half-cell units yields a complete full-cell stack, as depicted in Figure 2B. Driven by bidirectional transmembrane salinity gradients, cations selectively permeate negatively charged membranes, whereas anions migrate across positively charged counterparts, generating a cumulative diffusion potential between the terminal electrodes. In contrast to half-cell devices, full-cell architectures simultaneously capture charge fluxes from both cations and anions, delivering a theoretical energy conversion efficiency approaching 100%. Accordingly, full-cell membrane stacks are promising candidates for practical large-scale osmotic power generation.

### 2.2. Nanofluidic Ion Sieving Originates from EDL Overlap

**Figure 3:**
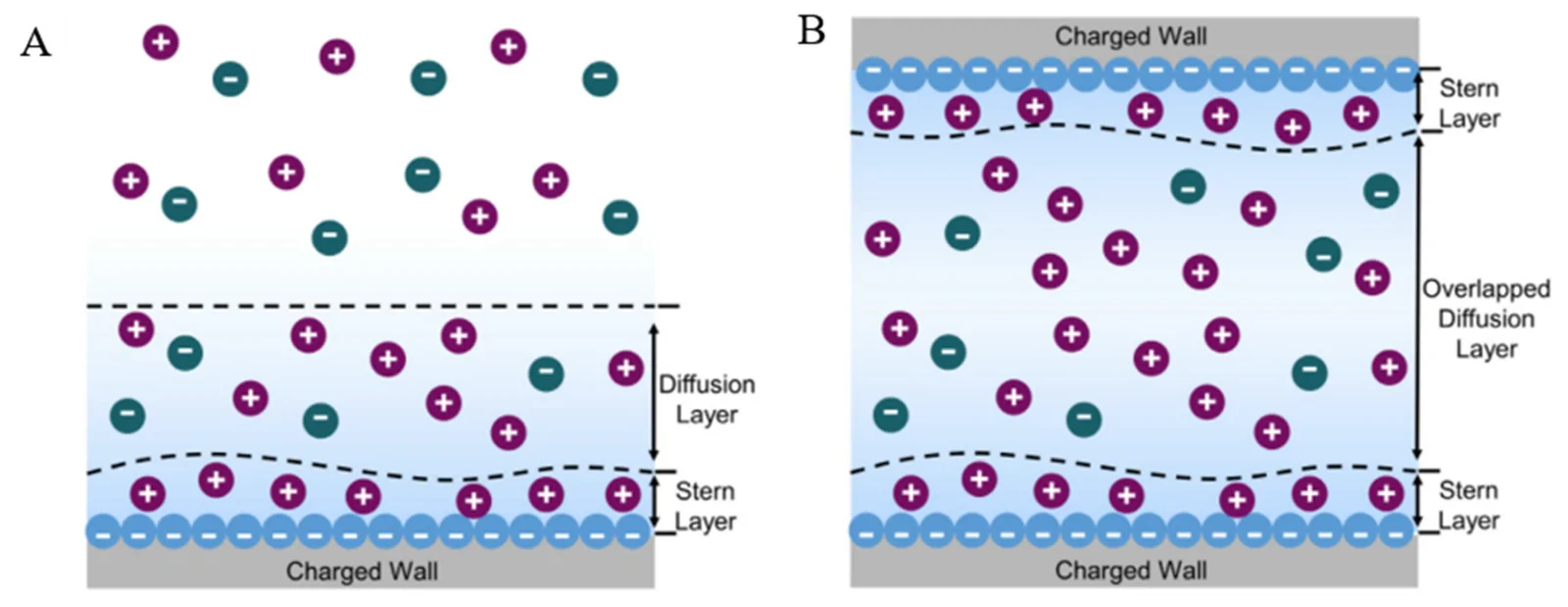
(**A**) Schematic representation of the electrical double layer (EDL) established at the interface of a negatively charged wall. (**B**) Illustration of ion permselectivity inside negatively charged nanochannels. If the characteristic size of nanochannels is comparable to the Debye screening length, co-ions are excluded via electrostatic repulsion, whereas counterions are allowed to transport through the nanochannels [35].

Ion transport in conductive polymer (CP)-based nanofluidic membranes is dominated by nanoscale electrostatic confinement [27,28,29,30], redox-active surface charges [31,32], and coupled electron–ion conduction [33,34,35]. Narrow nanochannels induce overlapping electrical double layers, enabling selective counterion permeation and multivalent ion rejection governed by Poisson–Nernst–Planck theory. Dual transport pathways coexist: low-resistance surface sliding along conductive CP skeletons and size-selective diffusion in hydrated interstitial pores. Redox doping reversibly tunes surface charge density to manipulate ion flux, whereas boundary-layer concentration polarization distorts the effective salinity gradient and suppresses power output in RED systems. These transport behaviors are governed by the electric double layer, whose thickness directly dictates the ion sieving capability of charged nanofluidic membranes (Figure 3A). When the nanochannel diameter is comparable to the EDL thickness (1–10 nm in dilute aqueous electrolytes), EDL overlap occurs inside confined channels. The surface charge on channel walls repels co-ions with identical charges and attracts counterions, forming exclusive counterion-transport pathways, which is the essential origin of nanofluidic ion permselectivity (Figure 3B). Driven by transmembrane salinity gradients, counterions spontaneously diffuse from the concentrated compartment to the dilute side, generating stable transmembrane diffusion potential and continuous ionic current.

Distinct from conventional polyelectrolyte-modified nanochannels with permanent static charges, the redox-active conjugated backbones of CPs support real-time charge regulation via external stimuli [36,37,38,39,40,41]. For PANI nanochannels, protonation in acidic environments induces positive surface charges for cation selectivity, while deprotonation under alkaline conditions switches the surface to negative charge for anion selectivity [42,43,44,45]. Reversible ion selectivity switching can be realized merely by adjusting solution pH, which cannot be achieved by traditional charged polymer channels.

### 2.3. Energy Conversion Efficiency

Osmotic energy harvesting relies on the ion permselective transport enabled by ion-selective membranes. The energy conversion efficiency of such systems is evaluated by comparing the recoverable electric energy with the theoretical free energy stored within the salinity gradient. For membrane-based reverse electrodialysis devices, the peak power density (*P*_max_) is defined by the following formula:

(2) $$P_{\max}=\frac{E_{\mathrm{osm}}^{2}}{4R_{m}}$$

where *E*_osm_ and *R*_m_ correspond to the transmembrane potential and the intrinsic membrane resistance, respectively [46,47]. Specifically, *E*_osm_ equals the Nernst potential established across the membrane, expressed as follows:

(3) $$E_{\mathrm{osm}}=S\frac{\mathrm{RT}}{\mathrm{zF}}\frac{c_{H}}{c_{L}}$$

where *S* denotes membrane permselectivity, while *C*_H_ and *C*_L_ represent the molar salt concentrations in the high-salinity and dilute feed solutions, respectively [48].

Connecting the membrane device to an external load resistance *R*_L_ constitutes another common approach to measure the peak power density. The maximum power density P_max_ is achieved under the impedance matching condition where the membrane intrinsic resistance *R*m approximates *R*_L_, which can be formulated as follows [49,50]:

(4) $$P_{\max}=I_{\mathrm{osm}}^{2}R_{L}$$

As discussed above, half-cell setups are widely utilized to characterize the ion-transport performance of ion-selective membranes for osmotic energy harvesting owing to their compact setup and straightforward operation. For half-cell devices equipped with cation-selective membranes, the energy conversion efficiency at peak output power can be simplified to the following equation:

(5) $$\eta_{\max}=\frac{1}{2}(t_{n}-1)$$

where *t*_n_ denotes the cation transference number [51]. This relation offers a convenient method for the preliminary assessment of osmotic energy conversion performance.

In practice, half-cell configurations cannot accurately reflect the true full-scale energy conversion efficiency due to multiple interfering factors encountered in practical engineering scenarios, including operational energy dissipation inside devices and charge polarization induced by enlarged test areas [52]. Accordingly, technological research can be divided into fundamental mechanistic investigations and engineering optimization tasks. Half-cell platforms serve as an efficient and facile tool for uncovering underlying transport principles, and they are indispensable for developing high-performance separation membranes.

## 3. Fabrication of Nanostructured Conductive Polymer-Based Membranes and Their Ion Transport Behaviors

### 3.1. In Situ Electrochemical Polymerization

**Figure 4:**
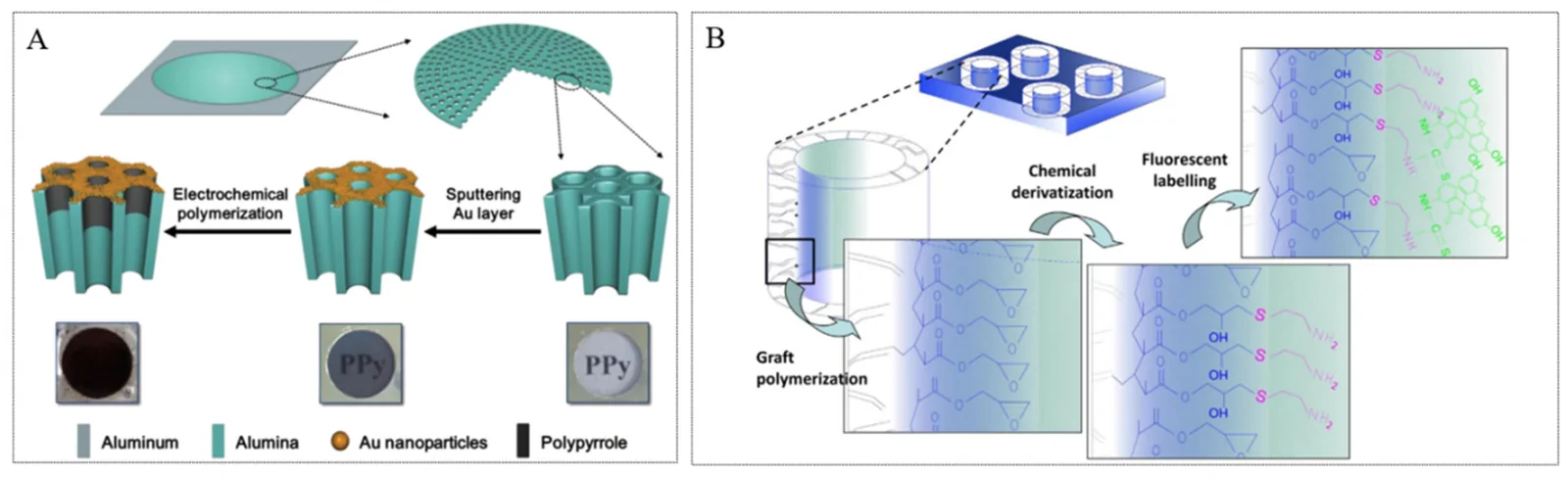
Fabrication of a conductive polymer-based nanofluidic membrane. (**A**) Schematic of the synthetic route toward PPy@anodic alumina hybrid nanochannels, paired with photographs recorded at every fabrication step [58]. (**B**) Illustration depicting grafting, chemical derivatization, and labeling processes of nanochannels fabricated from track-etched PET sheets [59].

Electrochemical polymerization serves as a dominant in situ fabrication strategy for conductive polymer (CP)-based nanofluidic membranes. It is commonly implemented using a standard three-electrode electrochemical cell with potentiostatic, galvanostatic, or cyclic voltammetry modes to trigger the oxidative polymerization of pyrrole, aniline, and EDOT monomers within nanoporous template substrates such as AAO and PET nanochannels. In situ electrochemical polymerization is the most mature laboratory preparation method. Porous template substrates (e.g., anodic alumina (AAO), track-etched PET nanochannels, and porous cellulose films) act as working electrodes, and pyrrole/aniline monomer solutions undergo constant potential or constant current polymerization to form uniform CP coatings on the inner wall of nanochannels [53,54,55,56,57,58,59]. Inspired by the asymmetric structure and stimulus-responsive ion transport of biological ion channels, Zhang et al. fabricated asymmetric PPy/Al_2_O_3_ organic-inorganic hybrid nanochannels by sputtering a gold conductive layer on one side of alumina nanopore arrays, followed by in situ deposition of polypyrrole (PPy) via electrochemical polymerization [58]. The dual pH- and light-modulated ion transport behaviors of the hybrid nanochannels were systematically investigated. pH variations triggered the protonation and deprotonation of alumina and PPy, leading to a discontinuous distribution of surface charge density and polarity inside the nanochannels. This spatial variation induced pH-dependent ionic–current rectification, with the rectification ratio significantly affected by the pore size and electrolyte concentration. The Poisson–Nernst–Planck (PNP) theoretical model reproduced the experimental rectification trends. At pH 6.8, alumina and PPy were positively charged, with PPy bearing more positive charges due to its high polymerization degree. Light excited PPy electrons from HOMO to LUMO at −2.3 eV (vs. NHE). The Nernst equation Φ = −0.059 pH gave a hydrogen reduction potential of −0.4 eV (vs. NHE) at pH 6.8. Photogenerated electrons reacted with electrolyte protons to form H· radicals, leaving holes that raised the positive charge density of the PPy layer in hybrid nanochannels. The synergistic effect of light irradiation and protons generated photogenerated holes on PPy and elevated the surface positive charge density of the nanochannels, realizing reversible light-regulated ion flux, while low pH conditions amplified such light-responsive ionic currents. Integrating the outstanding optoelectronic properties of conductive PPy and the high mechanical robustness of alumina membranes, this hybrid system exhibited favorable reversibility, stability, and stimulus sensitivity, offering a novel composite platform for the construction of smart nanochannel devices. Polymerization duration precisely controlled the coating thickness, and dopants including PSS and HCl could be introduced synchronously during polymerization to tune surface charge properties (Figure 4A). Espinoza et al. utilized residual active sites on swift heavy ion-etched PET films to initiate in situ graft polymerization of glycidyl methacrylate (GMA) on the inner walls of nanochannels [59]. The nanopore functionalization steps included chemical derivatization and fluorescent labeling. Epoxy groups anchored on grafted poly(glycidyl methacrylate) (polyGMA) chains were functionalized with cysteamine and subsequently tagged with fluorescein. A Nanodrop 3300 fluorometer was employed to directly detect the fluorescence of the treated membrane foils, and the fluorescence intensity was quantified in relative fluorescence units (RFU). The grafted polymers were fluorescently labeled with fluorescein isothiocyanate (FITC), and fluorescence spectroscopy, fluorescence microscopy, and confocal microscopy were utilized for visual qualitative observation and quantitative analysis of grafting behaviors (Figure 4B).

### 3.2. Intercalated Heterostructure In Situ Growth

Intercalated heterostructure in situ growth is a powerful strategy for constructing high-performance conductive polymer-based composite nanochannels for osmotic energy conversion. Taking layered substrates including MXene, V_2_O_5_, and MOF nanosheets as interlayer hosts, CP molecular chains such as PANI and PPy can be uniformly inserted into interlayer voids via controlled in situ polymerization. This synthetic route enables the formation of intimate inorganic–organic heterointerfaces, which effectively accelerate charge separation and transfer. Meanwhile, the intercalation process expands the original interlayer spacing, generating continuous vertical ion-transport pathways and alleviating the compression of electric double layers in hypersaline environments. Different from simple blending or solution casting, the in situ intercalated heterostructure avoids severe agglomeration of CP fillers and maintains unbroken conductive networks throughout the membrane. Benefiting from the synergistic effect between layered inorganic skeletons and redox-active conjugated polymers, the obtained composite membranes exhibit outstanding ion selectivity and transmembrane ion flux, showing promising prospects for industrial brine osmotic power harvesting in reverse electrodialysis devices.

**Figure 5:**
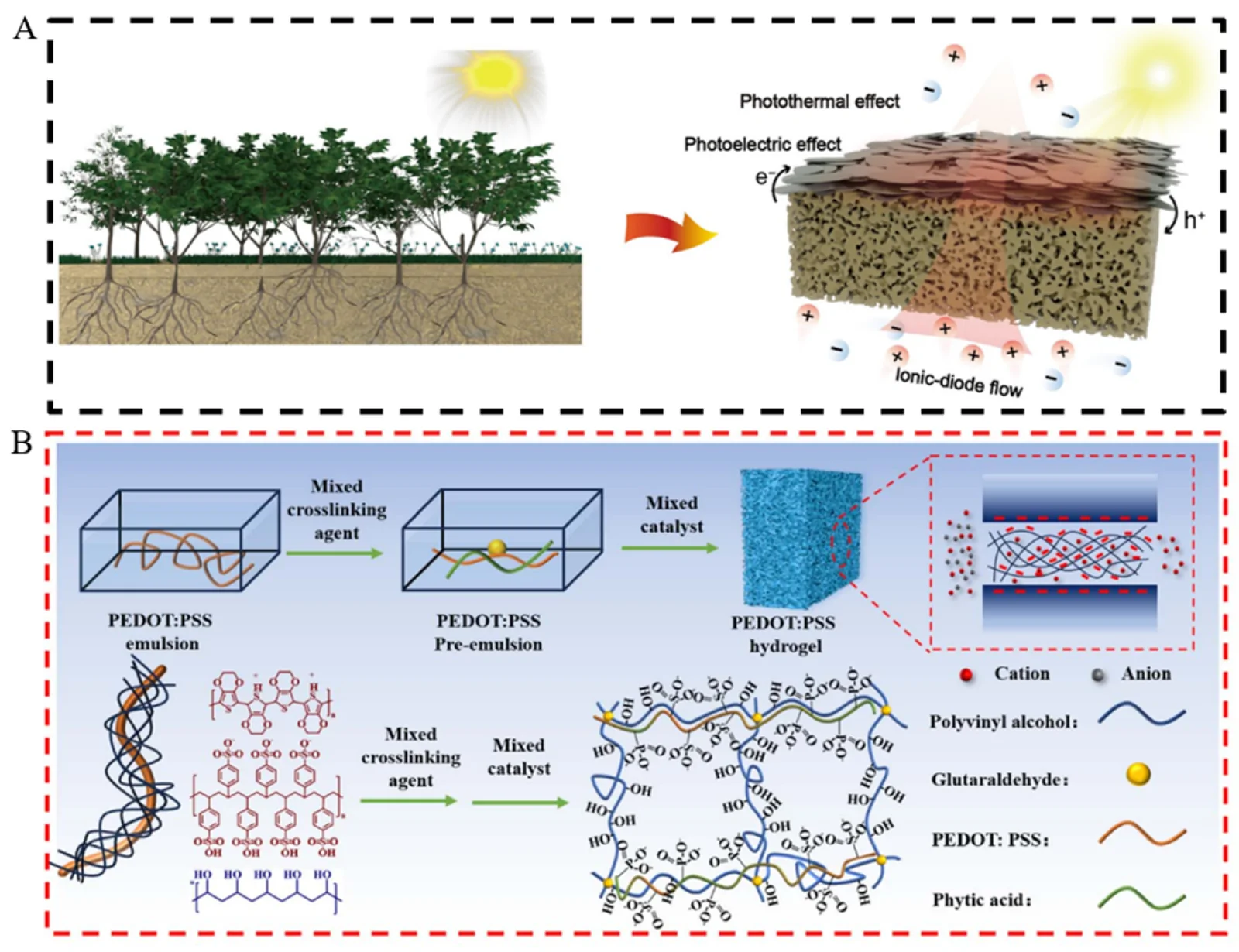
Preparation of a conductive polymer-based heterogeneous nanofluidic membrane. (**A**) Schematic depiction of the heterogeneous SPPO/PANI membrane, consisting of a sulfonated poly(2,6-dimethyl-1,4-phenylene oxide) (SPPO) matrix and a polyaniline (PANI) layer [61]. (**B**) Fabrication of 3D porous poly(3,4-ethylenedioxythiophene)–poly(styrenesulfonate) (PEDOT:PSS) hydrogel for osmotic membranes [62].

Conventional solid-state nanochannel devices suffer from critical drawbacks: the electrical double layer thickness shrinks drastically in high-ionic-strength (high-salinity) environments, leading to severe attenuation of ionic–current rectification. In addition, most such devices require complicated fabrication procedures and only operate under mild pH and low-salinity conditions, making them incompatible with harvesting osmotic power from high-salinity resources such as acidic hypersaline wastewater and salt lakes. Polyaniline (PANI), a conductive polymer with tunable surface charge responsive to both pH and redox stimuli, represents an ideal candidate for nanochannel modification; however, previous PANI functionalization approaches relied on multi-step complicated processes including metal sputtering and electropolymerization. Laucirica et al. [60] developed a facile one-step chemical modification strategy to coat polyaniline (PANI) only on the tip side of bullet-shaped ion-track-etched PET single nanochannels, constructing asymmetric PET/PANI nanofluidic diodes that exhibited outstanding pH- and redox-tunable ionic rectification even under hypersaline conditions up to saturated KCl. The PET/PANI membrane efficiently harvested salinity gradient osmotic power in acidic high-salt solutions with a maximum output power of 15 pW under a 1000-fold concentration gradient, breaking the limitation that traditional nanochannel devices lose rectification performance at high ionic strength. Heterostructured conductive polymers can construct dual-conductive nanofluidic channels with strong ionic rectification and hypersalinity tolerance, which effectively reduces membrane internal resistance and boosts the output power of osmotic power generation. Inspired by plant growth and photosynthesis, Zhang et al. developed a delicate heterostructure consisting of a sulfonated poly(2,6-dimethyl-1,4-phenyleneoxide) (SPPO) matrix and an in situ deposited polyaniline (PANI) layer for a high-efficiency osmotic–solar energy conversion system [61] (Figure 5A). This multiscale heterogeneous SPPO/PANI membrane fabricated via in situ aniline polymerization integrated a Janus asymmetric architecture and a polyaniline semiconductor heterojunction. This combination realized ionic–diode-rectified ion transport and light-driven ion pumping via the synergistic photothermal and photoelectric effects of PANI. Owing to its geometric, electrostatic and chemical multi-asymmetric features, the membrane achieved prominent unidirectional ion transport while alleviating concentration polarization, yielding an outstanding osmotic power density of 12.6 W m^−2^ under a 50-fold salinity gradient. Zhu et al. fabricated a low-cost and mechanically flexible, 3D porous negatively charged poly(3,4-ethylenedioxythiophene)–poly(styrenesulfonate) (PEDOT:PSS) hydrogel membrane through a one-step chemical–physical crosslinking method, which realized space-charge–controlled selective cation transport validated by multiple characterizations and theoretical simulations [62]. This PEDOT:PSS hydrogel membrane achieved a high osmotic power density of 6.26 W m^−2^, surpassing the commercial standard of 5 W m^−2^, and could be assembled into series devices to charge supercapacitors, offering a feasible strategy for large-scale osmotic energy harvesting and wearable bioenergy applications.

### 3.3. Electrospinning Nanofiber Membrane Fabrication

**Figure 6:**
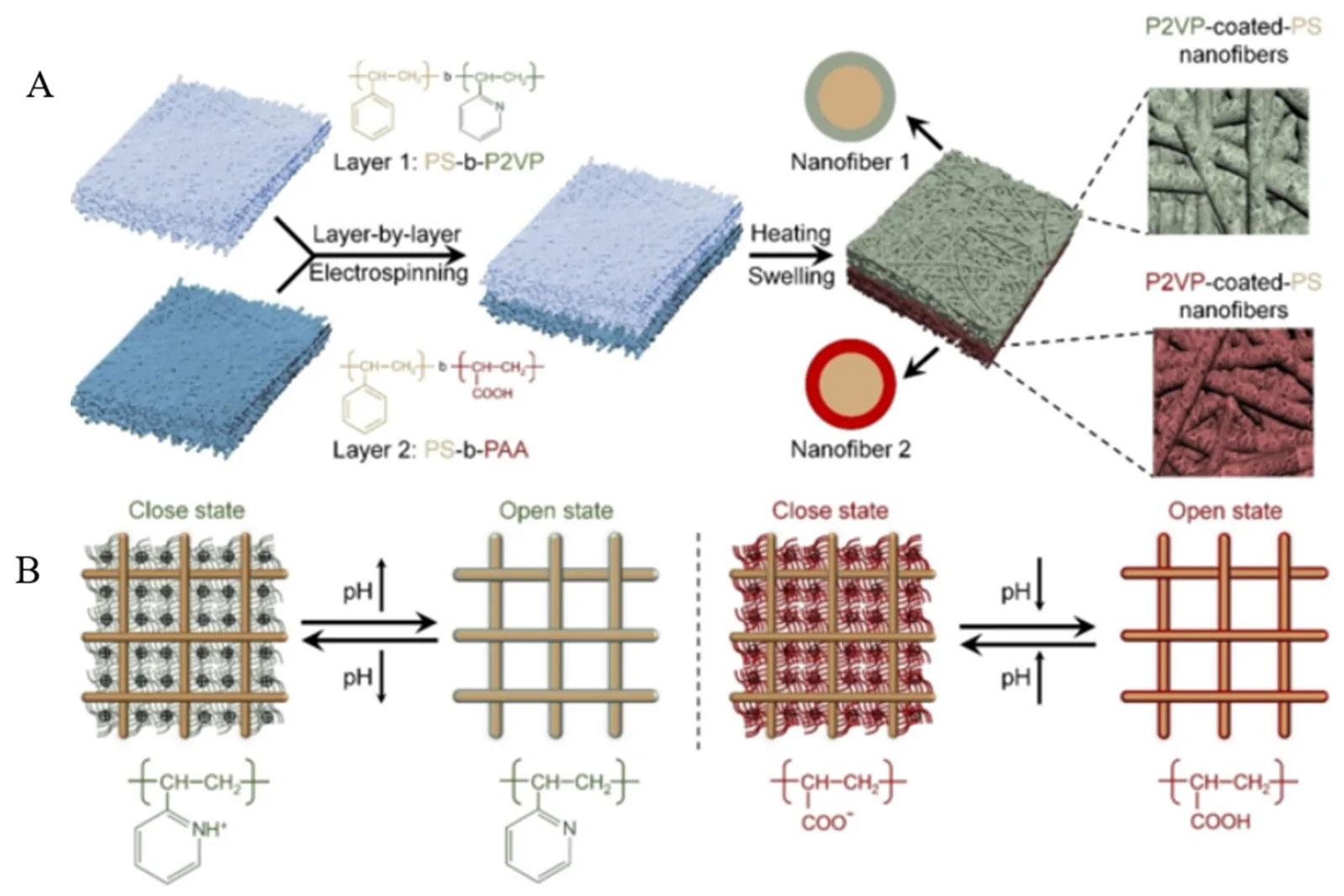
(**A**) Construction of a heterogeneous nanofiber membrane via electrospinning [64]. (**B**) Schematic of a single-layer PS-b-P2VP/PS-b-PAA nanofiber network. The chain conformations of P2VP and PAA are tunable via pH variations, reversibly switching the inter-fiber stacking pores between open and closed states.

Electrospinning technology produces three-dimensional interconnected CP nanofiber networks, generating abundant inter-fiber nanofluidic pores. Electrospun membranes feature high porosity and large specific surface areas, which significantly increases transmembrane ion flux. Post-doping treatment can further optimize membrane electronic conductivity, representing a promising route for flexible large-area osmotic membrane preparation. Rapidly polymerized electrospun PANI nanofibers maintain stable porous structures during long-cycle operation, which is favorable for continuous industrial manufacturing. Conductive porous materials with high porosity hold remarkable promise for energy management due to their efficient flow transmission and effective charge transport. Wang et al. developed a bridged conductive nanofibrous membrane (BCNM) by linking polypyrrole-coated nanofibers via self-assembled polypyrrole nanowires [63]. This dual-structured network enabled the construction of uninterrupted conductive pathways while maintaining multiscale porous channels, which synergistically integrated air ventilation, particulate interception, and electrothermal disinfection to realize multifunctional all-in-one air purification. This rational structure–function design enabled high-efficiency air purification, providing a universal template for next-generation conductive porous materials in biomedicine, environmental restoration, and energy production. Wen’s group reported heterogeneous electrospun nanofibrous membranes fabricated via sequential layer-by-layer electrospinning of PS-b-P2VP and PS-b-PAA, with a subsequent selective swelling step [64] (Figure 6). The nanofiber membranes exhibited effective ion-gating performance for tunable energy conversion. Pyridine and carboxylic acid groups anchored in the membrane created stable, unique ion-transport pathways, where nanochannels reversibly toggled between open and closed states in response to symmetric and asymmetric pH environments. The nanofiber membranes were applied to extract electric energy from osmotic gradients, delivering a maximum power density of 12.34 W m^−2^, and its ion-gating capability allowed the dynamic adjustment of system output power density. Such excellent performance renders nanofiber membranes promising candidates for smart nanofluidic devices, energy conversion systems, and water treatment technologies.

## 4. Conductive Polymer Materials for Osmotic Energy Conversion

### 4.1. Polypyrrole (PPy)-Based Membranes

**Figure 7:**
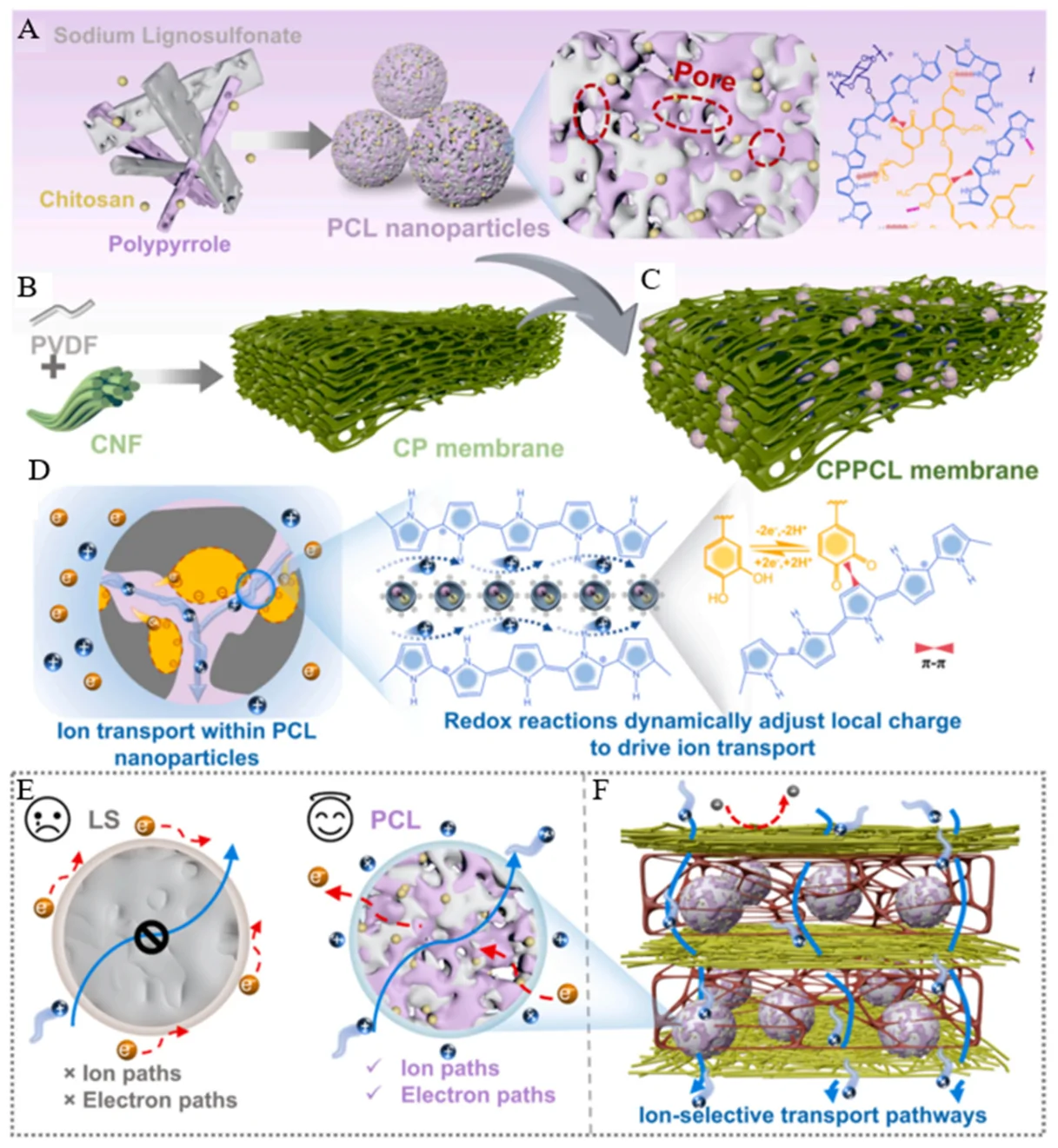
Schematic diagram depicting the fabrication route and operating mechanism of CPPCL composite membranes [65]. (**A**) PCL nanoparticles with hierarchical porous architectures are fabricated through noncovalent intermolecular interactions between sodium lignosulfonate (LS), chitosan (CS), and polypyrrole (PPY). (**B**) CP membranes are fabricated by mixing PCL nanoparticles with PVDF and cellulose nanofibers (CNFs). (**C**) The CP substrate membrane is composited with extra PCL nanoparticles to obtain the final CPPCL membrane. (**D**) The reversible quinone/hydroquinone (Q/HQ) redox reactions inside the PCL nanoparticles dynamically modulate the local surface charge field and accelerate the migration of ions and electrons. (**E**) Schematic diagram illustrating the electron and ion transport mechanisms inside LS and PCL nanoparticles: electrons travel along the interconnected PPY conductive network, whereas ions diffuse through the porous voids of PCL nanoparticles to accelerate ion migration. (**F**) The CPPCL membrane features a continuous 3D network architecture that constructs ion-permeable transport channels, which shortens ion migration distances and elevates overall ion transport efficiency.

PPy is the most extensively investigated conductive polymer for osmotic energy harvesting, featuring excellent aqueous stability, mild polymerization conditions, and superior inherent anion selectivity. Aiming at the universal bottleneck that ion selectivity and permeability cannot be simultaneously optimized in nanofluidic membranes for osmotic power generation, Zhang et al. innovatively fabricated redox-active PPy-chitosan-sodium lignosulfonate (PCL) nanoparticles with hierarchical pores, which were embedded into a three-dimensional network composed of cellulose nanofibers (CNFs) and PVDF to construct CPPCL composite membranes [65]. The conductive polypyrrole network enabled efficient electron transfer, while the reversible redox cycling of quinone/hydroquinone (Q/HQ) moieties in sodium lignosulfonate dynamically tuned the membrane surface charge density (Figure 7). Meanwhile, the inherent micro-meso hierarchical pores of PCL nanoparticles realized ion sieving and reduced mass transfer resistance. COMSOL (version 6.4) simulations verified that the three-dimensional interwoven channels provided shorter and smoother ion migration pathways compared to two-dimensional layered membranes. Under a 50-fold NaCl concentration gradient, the CPPCL membrane achieved a power density of 14.16 W/m^2^ without light irradiation, which was 15 times higher than pristine cellulose membranes. Benefiting from the synergy of the photothermal effect and the free-radical redox reactions of the PCL nanoparticles, the power density was further boosted to 21.72 W/m^2^ under unilateral illumination. In addition, the membrane possessed an outstanding tensile strength of 342.14 MPa and stably generated electricity in real seawater–river water systems. Its light-triggered bactericidal efficiency exceeded 98% via reactive oxygen species, endowing the membrane with superior anti-biofouling performance and long-term underwater service durability. This work delivers a novel design strategy and fabrication route for high-performance, multifunctional biomass-based membranes for blue energy harvesting. Zhang et al. fabricated photothermally responsive nanofluidic membranes using TEMPO-oxidized cellulose nanofibers (CNF), polypyrrole (PPY), and lignosulfonate (LS) [66]. LS regulated the uniform dispersion of PPY nanoparticles and improved the membrane’s surface charge, mechanical properties, and cation selectivity. The membrane delivered outstanding osmotic power generation performance under salinity gradients, and the trans-membrane temperature gradient generated by unilateral light irradiation significantly boosted the membrane’s output power density via the photothermal effect of PPY, offering a facile and scalable fabrication strategy for biomass-based photo-responsive membranes for blue energy harvesting.

### 4.2. Polyaniline (PANI)-Based Membranes

**Figure 8:**
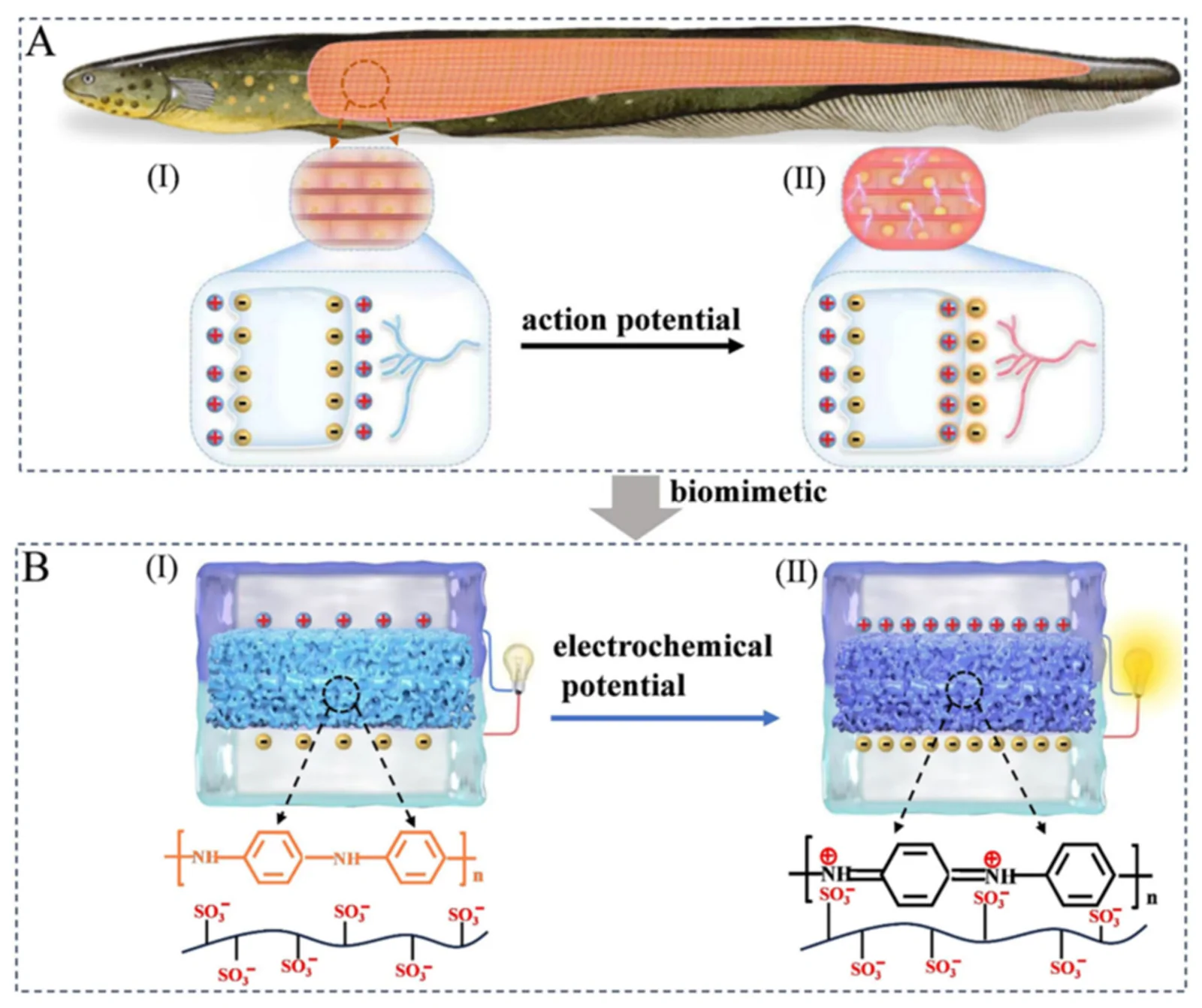
Schematic illustration of biomimetic electrically tunable osmotic energy harvesting inspired by the voltage regulation mechanism of electric eels [67]. (**A**) Voltage generation mechanism of electric eel electrocytes: in the resting state (**I**), the anterior and posterior membranes carry opposite potentials, producing a low output voltage; upon receiving motor neuron action potentials (activated state, (**II**)), the potential polarity of the posterior membrane is reversed to generate high-voltage discharge. (**B**) Electrically controlled energy conversion behavior of PSS-doped polyaniline (PSS-PANI) nanofluidic membranes: When PANI is electrochemically reduced (**I**), dedoping of PSS occurs on the neutral polymer backbone, lowering the matrix’s surface negative charge and yielding a low power output; in the oxidized state (**II**), high PSS doping density increases surface negative charge, which greatly boosts osmotic power generation.

PANI possesses unique electric-responsive charge reversal performance. Inspired by the bioelectrical regulation mechanism of electric eels, which generate controllable discharges through neural action potentials, Nie et al. fabricated PSS-doped polyaniline (PSS-PANI) conductive nanofluidic membranes on gold microgrid substrates via the electrochemical polymerization of aniline in sodium polystyrene sulfonate (NaPSS) electrolyte [67]. Polyaniline underwent reversible protonation and deprotonation under external potentials, accompanied by the doping and dedoping of PSS, which enabled reversible electrical modulation of the membrane’s surface charge polarity and cation selectivity (Figure 8). Characterizations including SEM, XPS, and FTIR verified that the pore size and continuity of the membrane could be tuned by adjusting the electropolymerization time, and the membrane obtained after 2 h of polymerization exhibited optimal ion-transport and osmotic energy-harvesting performance. Under a 50-fold KCl concentration gradient, the oxidized membrane achieved a cation transference number of 0.74 and a peak power density of 5.8 W/m^2^. In the reduced state, dedoping of PSS drastically reduced the surface negative charge and weakened cation selectivity, resulting in a much lower power density of only 1.3 W/m^2^. No obvious decay of output performance was observed after multiple redox cycles, demonstrating outstanding reversibility and stability. In addition, the output power rose with an increase in the salinity gradient and followed a descending order for K^+^, Na^+^, and Li^+^, revealing the influence of ion hydration radius on ion migration. This electrically tunable osmotic energy harvesting system fills the research gap in on-demand blue energy output regulated by electrical stimuli and offers a novel biomimetic design strategy for conductive polymer-based nanofluidic membranes with controllable osmotic power generation.

### 4.3. Poly(3,4-ethylenedioxythiophene): Poly(styrenesulfonate) and Polythiophene Derivatives

Poly(3,4-ethylenedioxythiophene)–poly(styrenesulfonate) (PEDOT:PSS) and various polythiophene derivatives serve as core conjugated conductive polymers suitable for salinity gradient power generation, including osmotic power and reverse electrodialysis. Featuring ultrahigh electronic conductivity and solution processability, PEDOT:PSS contains negatively charged groups on its PSS chains, which can construct ion transport channels with cation-sieving effects to efficiently capture cations under salinity gradients between seawater and freshwater and generate stable membrane potential. Polythiophene derivatives such as P3HT allow the modulation of band structures, hydrophilic pores, and ion affinity via molecular modification. These two materials can be used separately as ion-selective separation membranes or blended to fabricate integrated conductive electrode membranes, simultaneously realizing directional ion sieving and rapid electron conduction. This dual functionality addresses the drawbacks of traditional inorganic ion membranes, such as high cost, brittleness, and high internal resistance [68,69,70,71]. At present, researchers optimize the pore structure and carrier mobility of materials through doping modification, electrospinning pore formation, and nanocomposite strategies to improve the power density of salinity gradient power generation. Nevertheless, challenges remain, including insufficient stability in high-salinity environments and the trade-off between ion selectivity and electrical conductivity. Continuous development of novel thiophene-based functional polymers is critical to advance the large-scale application of low-cost flexible devices for salinity gradient energy harvesting.

**Figure 9:**
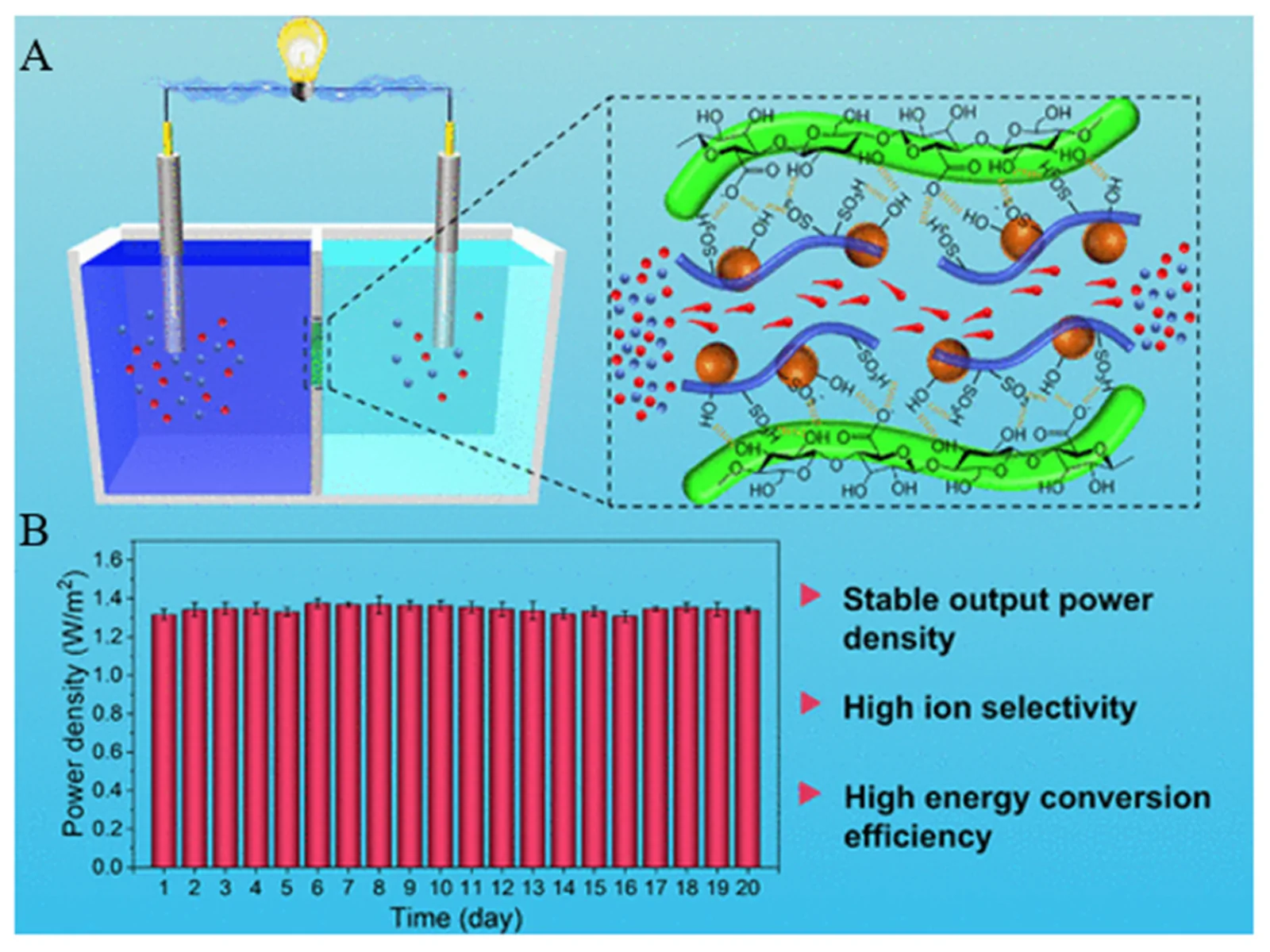
(**A**) Schematic representation of ion transport through the KL/TCNF-PEDOT:PSS membrane. (**B**) Stability test of salinity gradient power generation based on KL/TCNF-PEDOT:PSS membrane [76].

Cellulose nanofiber (CNF)-based nanofluidic membranes are promising candidates for osmotic power generation, yet their practical use is hindered by weak water resistance and low power output, arising from fiber swelling and entanglement in water [72,73,74,75]. Xu et al. constructed a composite nanofluidic membrane from TEMPO-oxidized cellulose nanofibers (TCNFs), kraft lignin nanoparticles (KL), and PEDOT:PSS [76]. The hydrophobic KL mitigated membrane swelling and fiber stacking to boost ion transport, while PEDOT:PSS introduced mixed ion–electron conduction and lowered internal resistance. Benefiting from the porous TCNF/KL framework interwoven with conductive PEDOT:PSS networks, the composite achieved an ionic conductivity of 0.02 S/cm under dilute pH 11 KCl solution. It reached an energy conversion efficiency of 36% and a cation selectivity of 0.92 under a 100-fold salt gradient, while delivering a peak power density of 1.37 W/m^2^ under 50-fold concentration difference. An 18-series stacked module lit bulbs and powered calculators (Figure 9). This work demonstrates that industrial kraft lignin is a low-cost filler for fabricating robust, high-performance nanofluidic membranes for green osmotic energy harvesting.

Conductive polymers such as PANI, PPy, and PEDOT:PSS serve as superior ion-selective membranes for salinity gradient power generation. Benefiting from their unique dual electron–ion conduction, their conjugated backbones cut membrane resistance and mitigate concentration polarization, while dissociable charged groups form space-charge layers to simultaneously achieve high ion flux and permselectivity [77]. Their surface charge is reversibly tuned via pH and redox reactions, allowing adjustable ion selectivity over a broad pH range and retaining obvious ionic rectification even in high-salinity acidic wastewater and salt lakes, which greatly broadens the applicable resources for blue energy harvesting [78,79,80,81]. Furthermore, facile fabrication strategies such as in situ polymerization and electrospinning easily construct asymmetric Janus, intercalated, or multiscale porous nanofiber structures with controllable thickness, pore size, and charge distribution to further optimize unidirectional ion transport. Their inherent electrochemical responsiveness enables real-time dynamic regulation of output power under external electric fields, and their lightweight, flexible characteristics support the development of miniaturized intelligent nanofluidic power devices. The structural design, fabrication methods, experimental testing conditions, and core energy harvesting indicators of state-of-the-art polyaniline, polypyrrole, and PEDOT:PSS nanofluidic membranes are summarized in Table 1. This table systematically sorts out key parameters, including membrane architecture, synthetic protocols, salinity gradients, and peak power density, enabling an intuitive horizontal comparison among various conductive polymer materials.

Inspired by natural nacre’s brick-and-mortar architecture, Chen et al. constructed MXene/PEDOT:PSS composite membranes through vacuum filtration, with the inserted PEDOT:PSS chains widening MXene interlayer gaps and raising the surface negative charge density to simultaneously strengthen mechanical robustness and tolerance to multiple organic solvents [82]. The composite membrane displayed strong cation-selective ion transport dominated by surface charge, and optimized samples generated an output power of 3216 ± 603 nW under a 2/0.001 M methanol–LiCl salinity gradient. Adding sodium hydroxide promoted the dissociation of negative charges inside the nanochannels, lifting the peak power to 6926 ± 959 nW with a power density of 55 mW m^−2^, and the device retained steady current output for four hours. The material achieved effective energy capture in diverse organic-salt mixed solutions, and a tandem stack of twelve membrane units was capable of powering a calculator, providing a biomimetic approach for recovering salinity gradient energy from waste organic solvents containing inorganic salts. Lee et al. fabricated thin pore-filled base anion-exchange membranes (PFAEMs) via UV polymerization on polyethylene porous substrates, and then modified the membrane surface with polypyrrole (PPy) and PPy/rGO composite layers through spin coating and thermal reduction treatment to suppress the uphill transport of multivalent ions in reverse electrodialysis [83]. The PPy/rGO coating introduced adjustable surface negative charges and a denser membrane surface structure, which enhanced electrostatic repulsion and the size-sieving effect against divalent anions without causing an obvious rise in overall membrane resistance. The optimized PPy/rGO-modified PFAEM achieved a monovalent anion selectivity over four times higher than commercial AMX membranes. When assembled into RED stacks, it effectively mitigated open-circuit voltage loss induced by multivalent ion crossover, delivering 5.43% higher peak power density than the commercial reference membrane. This work offers a facile surface modification strategy for boosting the energy harvesting efficiency of RED systems.

Light modulation boasts the unique advantages of remote, precise, and rapid control. Among photothermal materials, polypyrrole features high conversion efficiency and excellent stability, making it an ideal candidate. Zhang et al. constructed biomass-derived photothermal nanofluidic membranes (CNF-PPY-LS) via in situ oxidative polymerization of pyrrole within TEMPO-oxidized cellulose nanofiber networks using lignosulfonate as a dopant and dispersant [84]. Lignosulfonate effectively homogenized polypyrrole nanoparticles, elevated membrane surface charge density, improved mechanical strength and hydrophilicity, and delivered greatly enhanced ionic conductivity, cation selectivity, and osmotic energy output compared to undoped CNF-PPY counterparts. Under a 50-fold KCl salinity gradient without illumination, the optimized membrane achieved a power density of 1.87 W m^−2^, while the photothermal effect of polypyrrole created a cross-membrane temperature difference under light irradiation to synergize with the salinity gradient, further raising the peak power density to 2.38 W m^−2^ with reversible light-responsive ion transport behavior. The composite also exhibited outstanding long-term operational stability over one month, offering a facile, sustainable strategy for photo-tunable blue energy harvesting based on natural biomass materials. Traditional composite salinity power membranes face compatibility and fabrication drawbacks. Yu et al. prepared a single-component asymmetric polypyrrole film via simple oil–water interfacial polymerization [85]. It featured electroregulated ion transport, asymmetric wettability, and excellent cytocompatibility. Under a 0.5 M/0.01 M NaCl salt gradient, the membrane achieved a peak power density of 0.087 W/m^2^ and stable long-term osmotic energy output, demonstrating promise for implantable biomicro power supplies.

## 5. Improving Osmotic Energy Output Performance

Conductive polymers exhibit abundant external field-responsive behaviors, and their carrier concentration and microscopic aggregation structure can be reversibly regulated by electric fields [86,87,88,89,90,91,92] and light irradiation [93,94,95]. Applied bias voltage drives ion migration within the film layers and alters the doping level, simultaneously tuning electrical conductivity and optical absorption. Light irradiation at specific wavelengths induces the photoexcitation of conjugated chains or isomerization of side groups, generating reproducible photoelectric switching signals. The coupled regulation of multiple external fields can break through the performance limitations of single stimulation, offering design strategies for high-performance osmotic power generation membranes.

The charge density and internal resistance of nanofluidic membranes dominate their low ion permselectivity and ionic conductivity, both of which are pivotal metrics governing osmotic energy conversion. Notably, the charge density of a given material remains nearly constant under fixed operating conditions, whereas membrane internal resistance constitutes a lumped parameter encompassing multiple contributions. Membrane resistance falls into two categories: ohmic resistance originating from the membrane matrix and non-ohmic resistance derived from the diffusion boundary layer (DBL) [96,97,98,99]. Ohmic resistance stems from the resistance to ion transport across the membrane, whereas non-ohmic resistance corresponds to the ion entry barrier, which primarily originates from ion concentration polarization. Various strategies are available for modulating membrane resistive components. Based on a systematic literature review and our group’s previously reported experimental results, we categorize these osmotic power enhancement methods into two main routes: delicate membrane structural engineering and synergistic coupling with auxiliary functional technologies, which are elaborated in the subsequent sections.

### 5.1. Space Charge Regulation

In general, the surface charge density of nanochannels is intrinsically governed by surface functional groups capable of charge generation, including ionization, ion adsorption, ion exchange, ion dissolution, oxygen doping, and photoinduced electron–hole separation [100,101,102,103,104]. Accordingly, materials rich in charged functional groups can be adopted to elevate surface charge density. Nevertheless, restricted by existing fabrication techniques and material systems, tuning raw material compositions fails to further boost the surface charge density of nanochannels with fixed geometric dimensions and architectures. By contrast, grafting polymer brushes onto nanochannel interiors enables a dramatic enhancement of channel charge density. Moreover, charges are distributed uniformly across the entire nanochannel cavity, triggering a transition from a surface-charged regime to a space-charged regime. Numerous experimental and theoretical investigations across diverse nanofluidic membrane systems for reverse electrodialysis, including layered MoS_2_ nanosheets [102] and two-dimensional polymer membranes [105], have verified that the bulk space charge density within confined nanochannels can exceed conventional membrane surface charge density by several orders of magnitude under identical electrolyte operating conditions.

**Figure 10:**
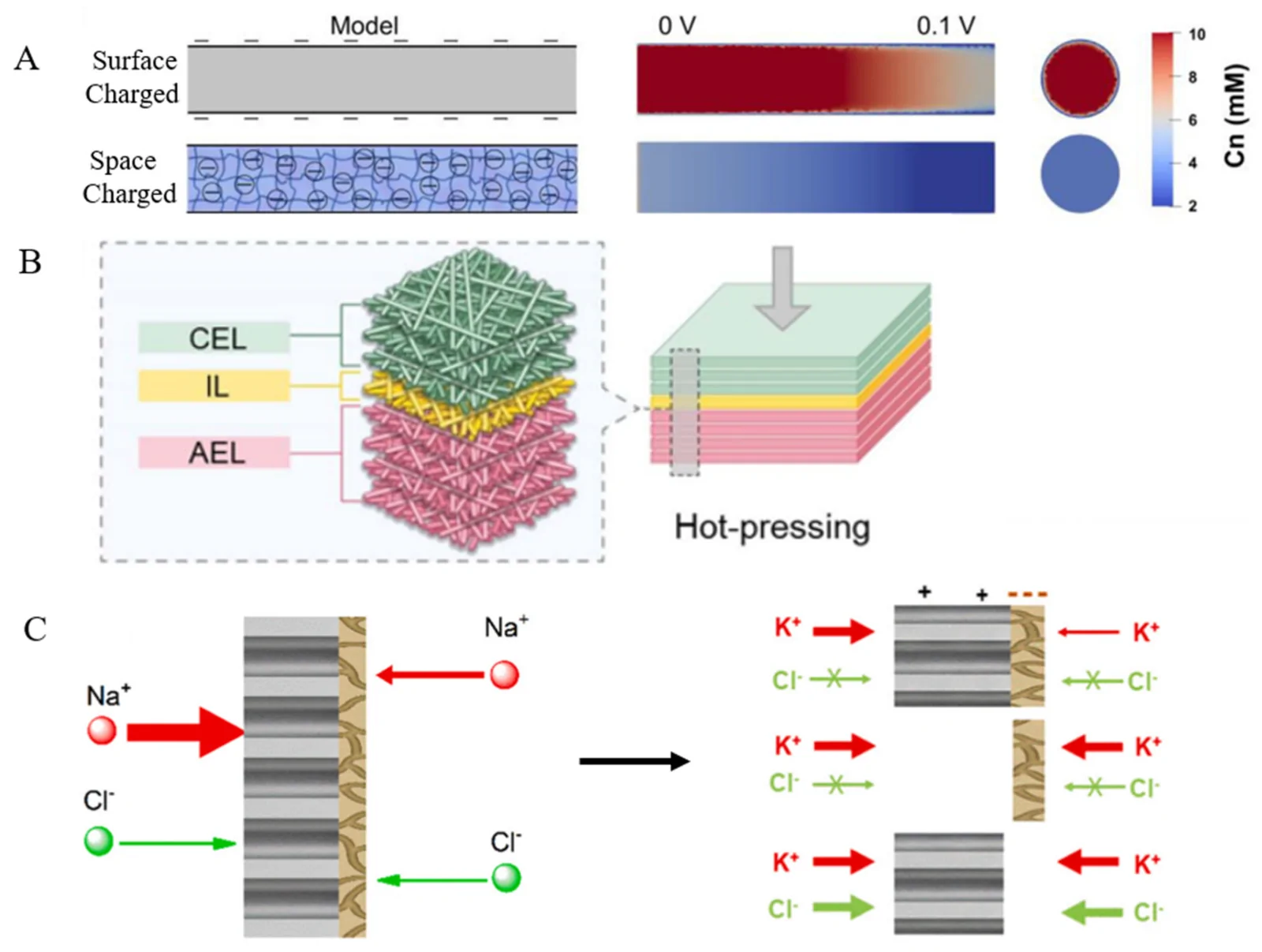
Strategies for membrane design toward high-performance osmotic energy harvesting. (**A**) Introducing space charges within nanochannels to boost ion permselectivity [106]. (**B**) Electrospun nanofluidic membrane fabricated by hot-pressing stacked electrospun mats, with the thickness regulated through the quantity of assembled CELs and AELs [107]. (**C**) Schematic diagram illustrating ion transport across distinct sides of asymmetric porous membranes under salinity gradients [108].

According to the Debye–Hückel equation, the effective range of the electric double layer spans several to tens of nanometers from the channel surface toward the bulk solution [106]. This characteristic length defines the spatial scope of electrostatic modulation over ion movement inside nanofluidic channels, and thus fundamentally governs the ion-selective transport critical to osmotic energy harvesting. If the total charge quantity remains unchanged, different charge distribution patterns will produce vastly different electrostatic influences on ion migration behavior within nanochannels. For negatively charged nanochannels (Figure 10A), the surface-charged model exhibits a high cation concentration limited only to the channel inner wall. Such localized electrostatic distribution cannot establish a dominating electrostatic field over the entire channel volume, which results in weak ion permselectivity and restricts the net ionic current available for power output. In space-charged nanochannels, electrostatic charges are uniformly distributed throughout the entire channel cavity rather than being confined to the inner surface. Consequently, the whole channel region exhibits prominent cation permselectivity, which favors asymmetric ion diffusion and improves the energy conversion performance. Drawing mechanistic support from these comparative analyses, the present study delivers rational, actionable strategies for designing high-efficiency membrane platforms applicable to osmotic power generation, providing instructive guidelines for the future development of bioinspired nanofluidic membranes.

### 5.2. Regulating the Thickness of Functional Coatings

The ohmic resistance provided by the membrane itself is directly associated with membrane thickness. Nanofluidic membranes face a fundamental performance inherent performance trade-off between low membrane resistance and superior ion permselectivity. Wang et al. used scalable electrospinning and precisely controlled hot-pressing to fabricate an electrospun nanofluidic membrane that simultaneously achieved both. A phytic acid and Fe^3+^ complex was utilized for the first time as a water dissociation catalyst [107]. Tuning the number of electrospun mats in the hot-pressing procedure enabled the production of electrospun BPMs with the thickness controlled from 40 to 90 μm (Figure 10B). Notably, decreasing the membrane thickness led to concurrent improvements in permselectivity and conductance. Reducing the number of electrospun mats subjected to hot pressing facilitated deeper penetration of the anion exchange layer into the interfacial layer, and the resulting thinner electrospun nanofluidic membranes attained reduced resistance alongside improved permselectivity. Zhang et al. developed a delicate heterostructure consisting of a sulfonated poly (2,6-dimethyl-1,4-phenylene oxide) (SPPO) matrix and an in situ deposited polyaniline (PANI) layer for a high-efficiency osmotic–solar energy conversion system [109]. A nanoscale ultrathin PANI functional layer was fabricated via controllable in situ polymerization. Benefiting from multiple asymmetries in geometry and surface charge, the membrane delivered efficient ionic–diode rectification, shorter ion-transport pathways, and drastically reduced membrane internal resistance. An ultrahigh osmotic power density of 12.6 W·m^−2^ was achieved under a 50-fold salinity gradient. This work offers a novel strategy for the structural design of ultrathin conductive polymer-based nanofluidic membranes and the coupled conversion of osmotic and solar energy. Duan et al. fabricated ultrastrong poly(p-phenylene-supported benzodioxazole) (PBO) nanofibers with a tunable thickness ranging from 0.81 to 3.88 μm via vacuum-assisted filtration [110]. They systematically verified a positive correlation between membrane thickness and ion transport resistance: the ultrathin architecture drastically shortened ion-migration pathways and effectively reduced areal resistance, delivering an ultrahigh power density of 40.2 W·m^−2^ under a 500-fold salinity gradient. Meanwhile, the rigid PBO backbone endowed the membrane with outstanding mechanical performance and long-term operational stability, with negligible performance decay after 40 consecutive days of power generation. This work fully demonstrates that membrane ultrathinning serves as an efficient strategy for simultaneously cutting internal resistance and boosting osmotic power output.

### 5.3. Construction of Heterogeneous Architectures

In reverse electrodialysis (RED) for salinity gradient power generation, concentration polarization leads to a massive loss of Gibbs free energy in the form of thermal dissipation, drastically reducing energy conversion efficiency. Bioinspired asymmetric nanofluidic membranes can generate ionic–diode rectification to suppress backward ion diffusion. Ding et al. fabricated dual-layer MXene heterogeneous ionic diode membranes with opposite surface charges via sequential vacuum filtration [111]. Benefiting from the asymmetric electric potential across the bilayer structure, the membrane achieved efficient unidirectional ion transport and alleviated concentration polarization. Experimental results verified that increased membrane thickness drastically raised transmembrane transport resistance, while the ultrathin 4 μm configuration balanced robust mechanical performance and low internal impedance. Under a 50-fold seawater–river water salinity gradient, a peak power density of 8.6 W·m^−2^ was realized, which further rose to 17.8 W·m^−2^ in a 500-fold hypersaline environment. Sun et al. synthesized two oppositely charged polymers derived from an identical polyethersulfone precursor and constructed bilayer Janus bipolar heterogeneous membranes by combining nonsolvent-induced phase separation and spin-coating techniques [112]. The homologous polymer backbone greatly improved the interfacial compatibility between the two membrane layers. Systematic experiments also confirmed that thicker membranes induced drastically elevated ionic transport resistance, and a thickness of 30 μm struck a balance between mechanical robustness and low internal impedance. The optimized membrane delivered a power density of 6.2 W·m^−2^ under a 50-fold salinity gradient, outperforming commercial Nafion 117. This research provides significant references for thickness and charge regulation of homologous ultrathin heterogeneous ionic membranes. Hou et al. constructed a charged porous asymmetric heterogeneous membrane composed of a 1.5 μm ultrathin sulfonated poly (ether ether ketone) (SPEEK) sieving layer and a macroporous anodic aluminum oxide (AAO) supporting layer [108]. Benefiting from geometric and charge asymmetry across the bilayer, which established an asymmetric transmembrane potential, the membrane enabled unidirectional cation transport and effectively alleviated concentration polarization (Figure 10C). The ultrathin dense layer delivered a high ion permselectivity of 90.8%, while the macroporous channels drastically reduced the areal resistance to 0.53 Ω·cm^2^. A peak power density of 12.5 W·m^−2^ was achieved under a 500-fold salinity gradient. This work offers a feasible strategy for developing ultrathin heterogeneous nanofluidic membranes that simultaneously possess high permselectivity and low internal impedance. Among various designs, Janus membranes integrating a charged nanochannel layer and an oppositely charged coating are the most widely adopted platforms for boosting osmotic power harvesting. The charged nanochannel layer is engineered to guarantee high ion permselectivity, while the oppositely charged layer alleviates ion concentration polarization through two distinct mechanisms. For one thing, the opposite surface charges strengthen electrostatic repulsion, lowering ion accumulation at the membrane–dilute solution interface. Moreover, abundant ions can permeate into the oppositely charged layer to endow the membrane with ion-storage capacity, which elevates the osmotic pressure difference and accelerates transmembrane ion migration [113]. In addition, Janus membranes exhibit prominent ionic rectification that favors unidirectional ion transport. By blocking backward ion diffusion across the membrane, the reverse dissipation of Gibbs free energy in the form of Joule heat can also be effectively suppressed [114].

## 6. Conclusions

Conductive polymer-based nanofluidic membranes integrate unique redox-tunable surface charge characteristics and excellent ionic–electronic dual conduction performance, perfectly matching the ion transport requirements of osmotic energy conversion. In recent years, remarkable progress has been achieved in the material design, preparation technologies, and performance optimization of PPy, PANI, and PEDOT derivatives and their heterostructured composite membranes. They deliver superior peak power density and ion permselectivity, demonstrating enormous application potential for large-scale blue energy exploitation. Future research should focus on intelligent responsive molecular design, hierarchical heterostructure channel engineering, low-cost scalable preparation technologies, and complete practical RED system integration. With continuous breakthroughs in materials science and membrane engineering, CP-based nanofluidic membranes are expected to become core functional materials for efficient salinity gradient energy utilization, making important contributions to the large-scale commercialization of clean marine renewable energy worldwide.

## Figures and Tables

**Table 1:** The salinity-gradient power generation performance of conductive polymers.

Conductive Polymer	Structural Configuration	Fabrication Method	Test Conditions	Peak Power Density	References
PANI	Heterogeneous membrane	Chemical oxidation polymerization	0.01/0.5 M NaCl	19.6 W/m^2^	[61]
PANI	Heterogeneous membrane	Electrochemical polymerization	0.01/0.5 M KCl	5.6 W/m^2^	[67]
PANI	Heterogeneous membrane	In situ polymerization	1000 fold	15 pW	[60]
PDOT:PSS	Continuous hydrogel network	Free–thaw crosslinking	0.01/0.5 M NaCl	6.26 W/m^2^	[62]
PDOT:PSS	Layer membrane	Filtration method	2/0.001 M methanol	6926 nW	[82]
PPy/RGO	PPy/RGO composite conductive membrane	In situ polymerization	0.5/0.01 M NaCl	0.408 W/m^2^	[83]
PPy/CNF	Nanofiber membrane	In situ polymerization	0.01/0.5 M KCl	2.38 W/m^2^	[84]
PPy	Asymmetric porous membrane	Interfacial vapor-phase self-assembly	50-fold NaCl gradient	0.087 W/m^2^	[85]

## Data Availability

No new data were created or analyzed in this study.
